# Sepsis Dysregulates Mitochondrial microRNA and Biogenesis in the Diaphragm but Not Limb Muscle

**DOI:** 10.3390/ijms27104222

**Published:** 2026-05-09

**Authors:** Luther Gill, Patricia Molina, Liz Simon

**Affiliations:** 1 Department of Physical Therapy, Louisiana State University Health Science Center, New Orleans, LA 70112, USA; 2 Department of Physiology, Louisiana State University Health Science Center, New Orleans, LA 70112, USA; pmolin@lsuhsc.edu (P.M.); lsimo2@lsuhsc.edu (L.S.)

**Keywords:** mitochondrial dysregulation, diaphragm muscle, microRNA

## Abstract

Diaphragm dysfunction that leads to respiratory failure is a significant clinical consequence of sepsis-induced critical illness. Diaphragm muscle weakness contributes to morbidity and mortality in these individuals in part due to impaired mitochondrial function. Restoring normal mitochondrial biogenesis is associated with improved survival and physical function. Therefore, identifying reliable biomarkers of mitochondrial dysfunction in diaphragm muscle will allow for more focused and targeted interventions designed to improve the morbidity of critically ill patients. We used a rodent cecal-ligation and puncture (CLP) model to mimic a moderate grade of sepsis. The diaphragm muscle was harvested from adult mice 48 h following CLP (*n* = 6) or a sham CLP procedure (*n* = 6). Our primary finding was that moderate grade CLP increases expression of mitochondria-associated microRNA in the diaphragm. Correspondingly, genes associated with mitochondrial biogenesis decreased. Our study provides evidence for sepsis-mediated dysregulation of mitochondrial homeostasis. This may play a role in diaphragm muscle dysfunction, respiratory failure, and difficult weaning from mechanical ventilation in sepsis-induced critical illness.

## 1. Introduction

Sepsis remains a leading cause of death worldwide. Defined as life-threatening organ dysfunction resulting from a dysregulated host response to infection and accounts for an estimated 11 million deaths globally [1]. This underscores the need for targeted therapies that move beyond supportive care. Increasingly, mitochondrial injury is recognized as central to sepsis pathobiology, driving metabolic failure through excessive reactive oxygen/nitrogen species, impaired mitophagy, disturbed dynamics, and membrane permeabilization, which together amplify inflammatory signaling and organ dysfunction [2].

Diaphragm fiber excitability and mitochondrial bioenergetics are markedly abnormal following sepsis. For example, sepsis reduces oxidative phosphorylation and depletes electron transport chain proteins in diaphragm muscle [3,4]. In preclinical models, sepsis causes sarcomeric disorganization, altered myosin expression, and reduced mitochondrial respiration in the diaphragm, alongside measurable decrements in contractile performance, in vivo diaphragm ultrastructure, and mitochondrial respiration within days, paralleling functional deficits measured in vivo; these findings align with clinical observations that respiratory muscle weakness complicates weaning and worsens outcomes [5]. These findings reinforce that the respiratory pump is an early and prominent target of sepsis-induced mitochondrial stress [5].

A major functional role of mitochondria is to supply a significant portion of adenosine triphosphate (ATP) needed to meet cellular energy needs via oxidative phosphorylation [2]. Indeed, diaphragm and limb muscle cellular energy metabolism and ATP production are altered during critical illness due to mitochondrial dysfunction [2,5,6]. Optimal mitochondrial function is determined by the appropriate coordination of key proteins that regulate electron transport, Krebs cycle function, and mitochondrial biogenesis [2,7]. Peroxisome proliferator-activated receptor gamma coactivator 1-alpha (PGC-1α), a transcriptional coactivator, is essential for maintaining mitochondrial biogenesis [2]. PGC-1α expression is reduced in diaphragm and limb muscle in critically ill patients, causing altered mitochondrial biogenesis [5,6]. This repair mechanism has been shown to compensate for mitochondrial damage and to play a major clinical role in survival following critical illness [2,7]. In addition to this, survivors contend with functional limitations, decreased force production, and skeletal muscle fatigue due to decreased ATP levels [6,8,9]. Exploring mechanisms of altered mitochondrial function in diaphragm muscle may elucidate therapeutic strategies that facilitate resumption of optimal diaphragm function in critically ill patients [5,7].

MicroRNAs (miRs) are a class of small noncoding RNAs that serve a regulatory role by inhibiting mRNA translation and modulating post-transcriptional gene expression [10]. They also play a significant role in regulating mitochondrial metabolism, apoptosis, and biogenesis, making them key contributors to mitochondrial dysfunction [11]. PGC-1α, a master regulator of mitochondrial biogenesis, is subject to multiple layers of regulation, including post-transcriptional suppression by microRNAs [10]. Although specific microRNAs such as miR-133, miR-23a, miR-494, miR-696, and miR-761 have previously been implicated in targeting PGC-1α, emerging research in mitochondrial microRNA biology continues to support their relevance in modulating mitochondrial function [11]. Moreover, physical activity has been shown to alter microRNA expression patterns in ways that promote mitochondrial biogenesis, consistent with decreased expression of mitochondrial-related miRs and increased PGC-1α signaling. This supports the notion that activity-responsive microRNA regulation of PGC-1α may represent a promising biomarker framework in the context of physical rehabilitation for sepsis-induced critical illness [10].

We selected the cecal ligation and puncture (CLP) mouse model because it closely reproduces the clinical and pathophysiological features of human sepsis, including profound mitochondrial impairment and diaphragm weakness—both major contributors to respiratory failure in critically ill patients. CLP-induced diaphragm and mitochondrial dysfunction have been well documented and mirror the respiratory muscle deficits observed in septic patients, underscoring the model’s translational utility [12,13]. We hypothesized that CLP-induced sepsis would preferentially suppress mitochondrial biogenesis gene expression in the diaphragm, but not in limb skeletal muscle, through the upregulation of specific microRNAs (e.g., miR-494 and miR-696). These microRNAs target key regulators of mitochondrial biogenesis, and their differential induction in the diaphragm may help explain why this muscle exhibits heightened vulnerability during sepsis. Understanding these diaphragm-specific molecular mechanisms is clinically important, as respiratory muscle failure is a major determinant of ventilator dependence, extubation failure, and mortality in septic patients. Identifying microRNA-driven disruption of mitochondrial homeostasis may therefore reveal new therapeutic targets aimed at preserving diaphragm function and improving outcomes in sepsis.

## 2. Results

### 2.1. Increased Cytokine Expression in the Diaphragm Following Cecal Ligation and Puncture

**Figure 1:**
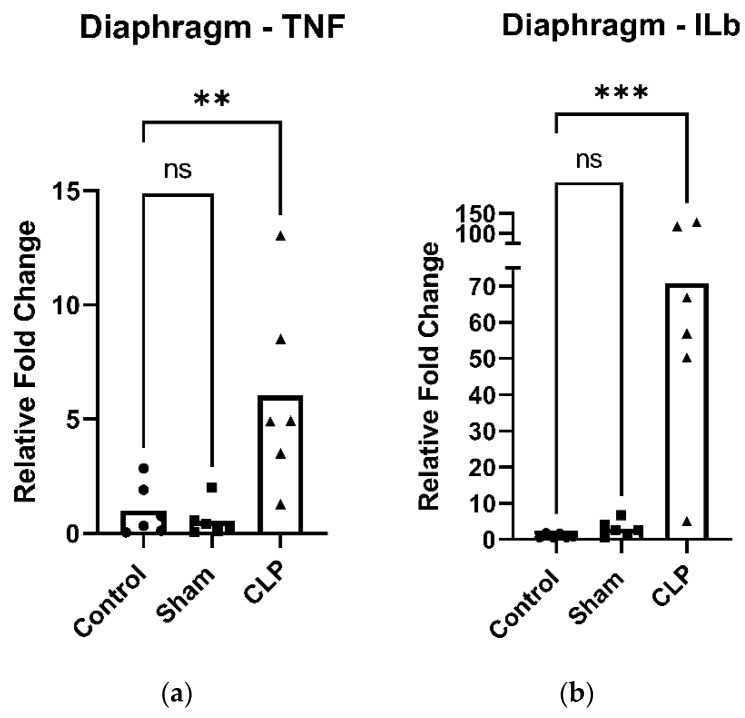
Inflammatory cytokine gene expression in the diaphragm 48 h following cecal ligation and puncture (CLP)-induced sepsis. (**a**) CLP significantly increased TNF expression compared to control. (**b**) CLP significantly increased ILβ expression compared to control. Data are expressed as means (bar) and individual data points for each group (five or six mice per group: control, sham, and CLP). ns: not significant, **: *p* < 0.01, ***: *p* < 0.001.

TNF (Figure 1a) and IL-1β (Figure 1b) expression were significantly elevated in the diaphragms of CLP animals compared with both control and sham groups. In contrast, inflammatory cytokine levels in limb muscle did not differ significantly among the groups.

### 2.2. Skeletal Muscle Atrophy Following CLP-Induced Sepsis

**Figure 2:**
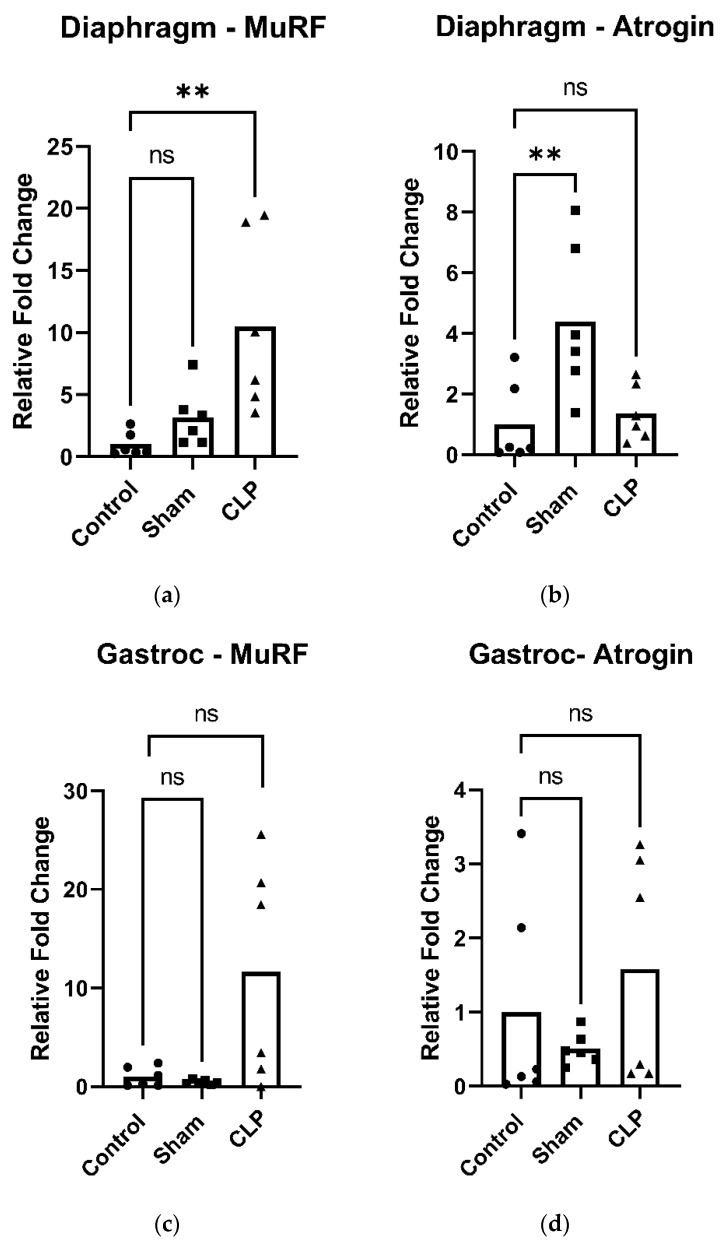
Gene expression of markers of muscle atrophy in the diaphragm 48 h following cecal ligation and puncture (CLP)-induced sepsis. (**a**) CLP significantly increased muscle RING-finger protein-1 (MuRF1) expression compared to control. (**b**) There was a significant increase in Atrogin-1 expression in the SHAM compared to control. There was no significant difference due to CLP. (**c**) There were no significant differences between the groups of MuRF1 expression in limb muscle 48 h following CLP-induced sepsis. (**d**) There were no significant differences between the groups of atrogin-1 expression in limb muscle 48 h following CLP-induced sepsis. Data are expressed as means (bar) and individual data points for each group (5 or 6 mice per group: control, sham, and CLP). ns: not significant, **: *p* < 0.01.

MuRF1 (muscle ring-finger protein-1) and atrogin-1 are two well-established markers of muscle atrophy [14]. In the diaphragm, MuRF1 expression was significantly increased in CLP animals 48 h after CLP-induced sepsis (Figure 2a), whereas atrogin-1 expression showed no change (Figure 2b). There were no significant differences in mRNA expression of MuRF1 and atrogin-1 in limb muscle at the 48-h time point (Figure 2c,d).

**Figure 3:**
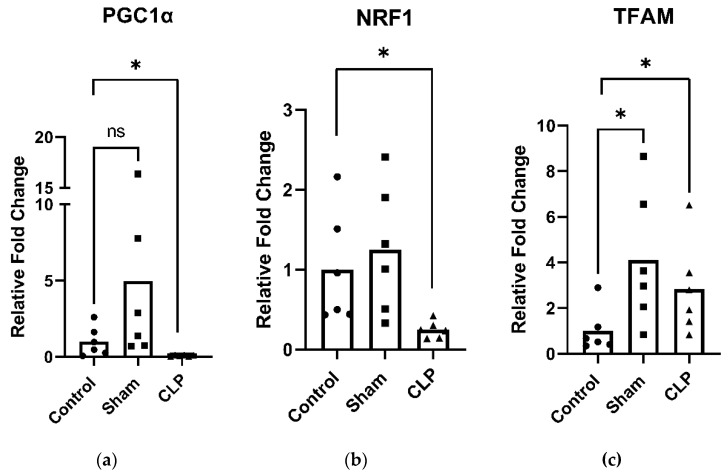
Gene expression of markers of mitochondria biogenesis in the diaphragm 48 h following cecal ligation and puncture (CLP)-induced sepsis. (**a**) CLP significantly decreased PGC1α expression compared to control. (**b**) CLP significantly decreased NRF1 expression compared to control. (**c**) CLP significantly increased TFAM expression compared to control. Data are expressed as means (bar) and individual data points for each group (5 or 6 mice per group: control, sham, and CLP). ns: not significant, *: *p* < 0.05.

**Figure 4:**
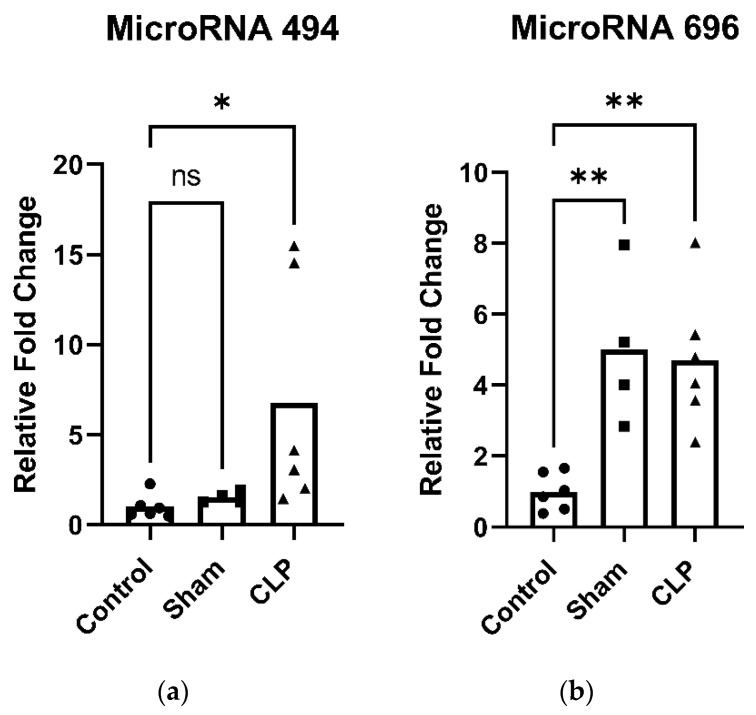
Expression of microRNAs associated with markers of mitochondria biogenesis in the diaphragm 48 h following cecal ligation and puncture (CLP)-induced sepsis. (**a**) CLP significantly increased microRNA 494 expression compared to control. (**b**) CLP significantly increased microRNA 696 expression compared to control. Data are expressed as means (bar) and individual data points for each group (five or six mice per group: control, sham, and CLP). ns: not significant, *: *p* < 0.05, **: *p* < 0.01.

PGC-1α—the master regulator of mitochondrial biogenesis—and its downstream transcriptional regulators, nuclear respiratory factor 1 and 2 (NRF1, NRF2) and mitochondrial transcription factor A (TFAM), are key mediators of mitochondrial biogenesis. In the diaphragm of CLP animals, expression of PGC-1α (Figure 3a) and NRF1 (Figure 3b) were significantly reduced compared to control, while TFAM (Figure 3c) was significantly increased compared to control. No differences in mitochondrial biogenesis-related transcription factors were observed in limb muscle. Additionally, mitochondrial-associated microRNAs miR-494 and miR-696 were significantly increased in the diaphragm of CLP animals compared to control and sham animals (Figure 4a,b).

## 3. Discussion

### 3.1. MicroRNAs Regulate Diaphragm Muscle Mitochondrial Function

MicroRNAs are a small class of non-coding RNAs approximately 22 nucleotides in length, and their regulatory networks play essential roles in skeletal muscle mitochondrial regulation, differentiation, proliferation, and apoptosis [15]. They modulate gene expression by binding to the 3′-untranslated region of target mRNAs, leading to either accelerated mRNA degradation or inhibition of translation [16]. MicroRNAs are responsive to physical activity and influence energy metabolism in both human studies [17,18] and animal models [19]. Mitochondria-associated microRNAs actively regulate mitochondrial function and may serve as reliable indicators of mitochondrial dysfunction [17].

PGC-1α, a master regulator of mitochondrial biogenesis, is known to be negatively regulated by several microRNAs, including miR-133 [20], miR-23a [21], miR-494 [21], miR-696 [22], and miR-761 [22]. Consistent with these regulatory interactions, we observed increased expression of miR-494 and miR-696 in the diaphragm of CLP mice.

### 3.2. Skeletal Muscle Mitochondria Dysregulation in Critical Illness

Critical illness induces profound dysregulation of skeletal muscle mitochondrial structure and function, leading to diminished muscle performance and heightened fatigue as a consequence of impaired ATP generation [2]. Experimental models demonstrate that intrinsic mitochondrial abnormalities can emerge as early as 18 h after the onset of cecal ligation and puncture (CLP)–induced critical illness. These abnormalities include distortion of both the inner and outer mitochondrial membranes and the development of mitochondrial swelling [23,24]. Such structural disruptions are closely associated with increased production of reactive oxygen species, inhibition of electron flow through the mitochondrial respiratory chain, and impaired oxidative phosphorylation, ultimately compromising mitochondrial ATP synthesis. The clinical relevance of mitochondrial dysregulation in critical illness is increasingly recognized. Impaired mitochondrial biogenesis and the inability to mount an adequate mitochondrial response are associated with poorer outcomes and reduced survival in critically ill patients [25]. Consistent with these observations, our findings show a significant reduction in the expression of PGC-1α, a key transcriptional regulator of mitochondrial biogenesis, at 48 h following CLP-induced sepsis. This decline was accompanied by decreased levels of NRF, further supporting the presence of sepsis-induced mitochondrial impairment.

### 3.3. Sepsis/Critical Illness: Impact on Diaphragm Muscle

Impaired mitochondrial biogenesis is increasingly recognized as a key contributor to diaphragm muscle dysfunction during critical illness [25]. In sepsis, mitochondria exhibit profound ultrastructural abnormalities, including swelling and disruption of both inner and outer mitochondrial membranes. Under normal conditions, mitochondrial biogenesis serves as a compensatory repair mechanism that restores mitochondrial number and function, thereby supporting cellular homeostasis and survival.

MicroRNAs are emerging as important regulators of mitochondrial biology in skeletal muscle, in part through their suppression of critical transcriptional mediators of mitochondrial biogenesis such as PGC-1α, NRF1, and TFAM. In our cecal ligation and puncture (CLP) model, we observed that 48 h after injury, expression of mitochondrial biogenesis markers were significantly reduced, coinciding with an upregulation of mitochondria-related microRNAs. These findings align with clinical observations demonstrating that mitochondrial repair responses are often insufficient during severe critical illness.

Importantly, an early induction of mitochondrial biogenesis (within 1–2 days of ICU admission) has been documented in skeletal muscle of patients who survive sepsis, but not in those who ultimately succumb. Moreover, septic non-survivors exhibit markedly lower ATP levels in vastus lateralis biopsies collected within the first 24 h of ICU admission, and skeletal muscle ATP content correlates strongly with eventual clinical outcome. Together, these data highlight impaired mitochondrial recovery as a central determinant of muscle dysfunction and mortality during critical illness.

### 3.4. Skeletal Muscle Response to CLP-Induced Sepsis: Diaphragm vs. Limb Muscle

Despite clear evidence of systemic infection in our model, we did not observe differences in gene or microRNA expression in limb muscle at the 48-h time point. The divergent responses between diaphragm and limb muscle may reflect the temporal limitations of the CLP model, which typically produces a severe but relatively short-lived septic insult, with substantial biological evolution beyond 48 h in many tissues [26]. Although both tissues are phenotypically skeletal muscle, they differ in embryological origin, anatomy, and developmental programs, which may shape downstream metabolic and stress responses [27]. Prior CLP and sepsis-focused studies of the diaphragm demonstrate mitochondrial dysfunction, structural injury, and contractile impairment, aligning with our observations [5,28].

Clinical and preclinical evidence indicate that the diaphragm undergoes rapid and pronounced atrophy or dysfunction in response to inactivity and/or mechanical ventilation, often on the order of hours to a few days [29,30]. For example, Levine et al. reported ~50% diaphragm fiber atrophy after 18–69 h of controlled mechanical ventilation in humans, with concurrent oxidative stress and proteolytic activation [31]. Serial ultrasound and cohort studies in ventilated ICU patients likewise show early diaphragm thinning or reduced thickness and functional decline, with important clinical implications [32,33]. Consistently, CLP and endotoxemia models reveal early diaphragm weakness and mitochondrial derangements [28].

At the onset of CLP-induced sepsis in rodents, the diaphragm exhibits significant oxidative/nitrosative stress and membrane injury that can persist for hours to days, while mechanical loading modulates the extent of damage [34]. In contrast, limb muscles may show different oxidative stress kinetics or magnitudes at comparable time points, highlighting muscle-specific redox responses [35]. Early mitochondrial dysfunction—spanning electron transport chain impairment and altered coupling—has been documented in the septic diaphragm and can be mitigated by mitochondria-targeted antioxidants in preclinical models [28,35]. Clinically, preferential diaphragm atrophy relative to peripheral muscles has been demonstrated in septic ICU patients using 3-D CT volumetry, with reduced diaphragm strength compared with non-septic controls [36].

Given these physiological and temporal differences, it is plausible that a sepsis model allowing a longer, sustained insult (e.g., modified CLP with prolonged survival support) would reveal limb-muscle microRNA changes that are not detectable at 48 h [26]. Collectively, evidence supports that diaphragm and limb muscles differ in their timing and biological responses to systemic infection—particularly in mitochondrial function, redox signaling, and injury susceptibility [2,37].

### 3.5. Clinical Implications and Future Directions

Critical illness caused by systemic sepsis is characterized by profound metabolic disturbances, the severity of which is strongly associated with increased morbidity and mortality [38,39,40]. Evidence from skeletal muscle biopsies of critically ill patients demonstrates reduced ATP levels, suggesting that mitochondrial function, cellular energy metabolism, and ATP production are impaired in skeletal muscle during critical illness [25].

Clinical laboratory biomarkers may enable earlier detection of skeletal muscle mitochondrial dysfunction and support the development of targeted therapies. Mitochondria-related microRNAs may serve as reliable indicators of mitochondrial impairment. In future studies, we will examine microRNAs in the serum of critically ill patients to evaluate their potential as biomarkers of diaphragmatic dysfunction and predictors of patient outcomes.

### 3.6. Study Limitations

Clinical management of sepsis routinely includes ongoing fluid resuscitation and timely administration of antibiotics when indicated. In contrast, commonly used preclinical models of sepsis—such as cecal ligation and puncture (CLP) or lipopolysaccharide (LPS) administration—typically demonstrate substantially higher mortality rates than those observed in the clinical population. Moreover, antibiotic therapy is often intentionally withheld in these models to better characterize underlying mechanisms of dysfunction. As a result, differences between clinical practice and experimental design may limit the direct translational relevance of these findings.

While numerous gene expression changes were detected in the present study, transcriptional alterations alone do not confirm a causal role in diaphragm muscle weakness. Follow-up studies that quantify corresponding protein expression and directly test whether these molecular changes contribute to impaired diaphragm function will be essential. Several aspects of diaphragm dysfunction have already been documented in the CLP model, including reductions in muscle force generation, impaired neuromuscular transmission, mitochondrial dysfunction, and diminished endurance capacity [41]. In the current data, changes in inflammatory markers (TNF-α, IL-1β) and the atrophy-associated E3 ligase MuRF1 are consistent with established pathophysiological responses in the CLP model. Additionally, prior work has shown that microRNA-696 targets PGC-1α—a master regulator of mitochondrial biogenesis [22].

A further limitation is that all measurements were obtained from whole-tissue diaphragm homogenates. This approach does not allow differentiation between changes originating from muscle fibers, infiltrating immune cells, fibroblasts, endothelial cells, or other resident cell populations. Consequently, the cellular source of the observed molecular alterations remains unclear.

Finally, diaphragm dysfunction is often characterized by reduced force generation during key respiratory behaviors such as tidal breathing, coughing, and forced expiration. Given the diaphragm’s exceptionally high mitochondrial density—required to sustain approximately 900 contractions per hour, we believe mitochondrial impairment resulting from systemic infection plays a major role in disrupting normal diaphragm performance. Future studies incorporating cell-specific analyses, mitochondrial functional assays, and direct measurements of contractile performance will help clarify the mechanistic links between systemic infection, mitochondrial health, and diaphragm weakness.

## 4. Materials and Methods

All experimental protocols were approved by the Institutional Animal Care and Use Committee at the Louisiana State University Health Sciences Center (LSUHSC) in New Orleans. Adult male (12 weeks—25 g) C57BL/6 mice (*n* = 6 per treatment group) were purchased from Charles River Laboratories, Wilmington, MA, USA), were singly housed during a week-long acclimation period in a controlled environment and for the entire study period.

### 4.1. Cecal Ligation and Puncture Model

All surgical procedures were performed under aseptic conditions. Mice were anesthetized in an induction chamber using isoflurane (3–4% in 100% O_2_), after which anesthesia was maintained via nose cone delivery of 2–3% isoflurane in 100% O_2_. The abdominal area was shaved and disinfected prior to surgery. A 1–2 cm midline laparotomy was performed to expose the peritoneal cavity. The cecum was gently exteriorized, and approximately 50% of its length below the ileocecal valve was ligated using a sterile 3.0 silk suture. Following ligation, a single through-and-through puncture was created midway between the ligation site and the cecal tip using an 18-gauge needle, consistent with a moderate-grade septic insult as defined in established CLP guidelines [12,13].

The selected ligation length (50% of total cecal length) produces a mid-grade sepsis model characterized by approximately 40% survival, with ~40% mortality occurring within 72 h and an additional ~20% mortality by 5 days. This level of severity provides a clinically relevant systemic inflammatory response while limiting excessive morbidity. The diaphragm undergoes rapid structural and functional alterations following systemic inflammation and reduced activity, as documented in both human and animal studies. Based on this evidence, tissues were collected 48 h after CLP to capture early sepsis-induced diaphragmatic dysfunction while minimizing confounding effects from late-stage septic deterioration.

Following cecal puncture, a small amount of fecal material was extruded to ensure patency, after which the cecum was returned to the abdominal cavity and the peritoneum, fascia, and abdominal musculature were closed using continuous running sutures. The skin was closed separately with interrupted sutures. After wound closure, all mice received 1 mL of sterile saline subcutaneously for fluid resuscitation and were allowed to recover from anesthesia under observation. Upon recovery, all animals regained full cage mobility and were physically capable of obtaining food and water ad libitum. In this model, the CLP procedure does not impair gross locomotor activity during the first 24–30 h post-surgery. When an animal demonstrated difficulty accessing food, a nutrient-rich gel was provided directly within the cage to ensure adequate intake. Sham animals underwent identical anesthesia, laparotomy, and wound-closure procedures but did not receive cecal ligation or puncture. Naïve control mice did not undergo any surgical manipulation and were not exposed to isoflurane anesthesia.

To confirm the establishment of sepsis, blood samples (50 µL) were collected from a subset of sham and CLP animals and plated on blood agar using a serial dilution approach. Plates were incubated for 24 h to permit colony formation. Robust bacterial growth was observed in samples from CLP mice, with a mean bacterial burden of 2.4 × 10^7^ cfu/mL at 24 h post-surgery, whereas no colony growth was detected in sham animals. Additionally, systemic inflammation was verified by quantifying expression of the key cytokines TNF and IL-1β, both of which are established markers of sepsis-associated inflammation.

### 4.2. Skeletal Muscle Harvest and Processing

Forty-eight hours after the CLP procedure, animals were euthanized, and diaphragm and lower limb muscles (gastrocnemius) were collected. The harvested muscles were rapidly frozen in liquid nitrogen and stored at −80 °C for subsequent molecular analyses. To evaluate the impact of moderate CLP on both diaphragm and gastrocnemius muscles, we quantified the expression of two well-established markers of skeletal muscle atrophy: MuRF1 (muscle ring-finger protein-1) and atrogin-1 [14].

### 4.3. Quantitative PCR for Gene Expression

Total RNA was isolated from diaphragm and gastrocnemius muscle tissues using the miRNeasy Mini Kit (Qiagen, Valencia, CA, USA) according to the manufacturer’s protocol. Complementary DNA (cDNA) was synthesized from 1 µg of total RNA using the QuantiTect Reverse Transcription Kit (Qiagen). Primers spanning exon–exon junctions were designed and synthesized by Integrated DNA Technologies (Coralville, IA, USA). Quantitative PCR (qPCR) reactions were prepared to a final volume of 20 µL using the QuantiTect SYBR Green PCR Kit (Qiagen) and DNase/RNase-free water. All reactions were performed in duplicate on a CFX96 Real-Time PCR Detection System (Bio-Rad Laboratories, Hercules, CA, USA). Gene expression was quantified using the comparative CT (ΔΔCT) method, with ribosomal protein S13 (RPS13) serving as the endogenous reference gene. Expression levels in experimental groups were normalized to corresponding control values. To assess the impact of cecal ligation and puncture (CLP) on mitochondrial function in diaphragm and gastrocnemius muscle, we evaluated mRNA expression of established markers of mitochondrial biogenesis, including PGC-1α, mitochondrial transcription factor A (TFAM), and nuclear respiratory factor-1 (NRF1).

### 4.4. Quantitative PCR for miRNA Expression

Expression levels of microRNAs (miRNAs) in skeletal muscle were quantified using individual TaqMan MicroRNA assays (Thermo Fisher Scientific, Waltham, MA, USA). Total RNA previously isolated for gene expression analyses served as the template. For each reverse transcription (RT) reaction, 10 ng of RNA were combined with the TaqMan MicroRNA Reverse Transcription Kit and miRNA-specific stem-loop primers (Table 1; Thermo Fisher Scientific, Waltham, MA, USA) following the manufacturer’s instructions. For quantitative PCR (qPCR), 1.33 µL of the resulting RT product was added to 18.67 µL of PCR reaction mix consisting of 10 µL TaqMan Universal PCR Master Mix II containing AmpErase uracil-N-glycosylase, 1 µL of the appropriate TaqMan miRNA assay reagent, and 7.67 µL nuclease-free water, yielding a final reaction volume of 20 µL. qPCR was performed on the CFX96 Real-Time PCR Detection System (Bio-Rad) under the following cycling conditions: 50 °C for 2 min and 95 °C for 10 min, followed by 40 cycles of 95 °C for 15 s and 60 °C for 1 min. Small nucleolar RNA U6 served as the endogenous control for normalization and was amplified in duplicate wells on each plate. Relative expression levels were calculated using the comparative threshold cycle (ΔΔCT) method. To assess the impact of cecal ligation and puncture (CLP) on mitochondrial-related miRNA expression in diaphragm and gastrocnemius muscle, we measured miR-133, miR-23a, miR-494, miR-696, and miR-761.

### 4.5. Statistical Analysis

One-way analysis of variance (ANOVA) was performed to assess differences in gene and miRNA expression among experimental groups with Tukey–Kramer honestly significant difference post hoc tests when appropriate. All statistical analyses were conducted using GraphPad Prism 9 (La Jolla, CA, USA). Statistical significance was defined as *p* < 0.05. Data are presented as mean ± SEM unless otherwise specified.

## 5. Conclusions

Alterations in cellular energy metabolism and ATP production are well-documented features of diaphragm muscle dysfunction in critically ill patients, arising in part from impaired mitochondrial repair and quality-control mechanisms. These mitochondrial repair pathways—which include biogenesis, mitophagy, and fusion–fission dynamics—are essential for maintaining mitochondrial integrity during physiological stress and have been strongly implicated in survival among patients in the intensive care unit (ICU). In the present study, CLP-induced sepsis significantly downregulated genes governing mitochondrial biogenesis, accompanied by increased expression of mitochondria-associated microRNAs known to negatively regulate these pathways. These findings suggest that mitochondria-related microRNAs may serve as clinically informative biomarkers of metabolic and bioenergetic insufficiency. Accordingly, characterizing the clinical trajectory of critically ill patients with underlying mitochondrial dysfunction using these microRNAs as indicators of morbidity and mortality could provide a foundation for future prospective investigations.

## Figures and Tables

**Table 1:** List of primers used.

Gene	Forward Primer	Reverse Primer
TFAM	TGGATGGCATGGGTTTAAG	TCACGTCTCTCCTGGATTT
PGC1A	ACCTCTCTCTCTCTCTCT	TCTCCCACCCAGATTCAA
PGC1B	GTGGTTGCTGGGAAAGATAA	CAGTGCTTAAAGGATGTAGGG
NRF1	GTGAAATAAGCCTCCCGATAG	GAGGCAGTTTAGACAGAATGG
NRF2	TTCATAGCAGAGCCCAGT	CAGGTCACAGCCTTCAATAG
ATROGIN	GAGTGGGAGAGGTGTAGAA	CTCTGGCCATGACCTAATATG
MURF1	CTTGAGGGCCATTGACTTT	GGTGTTCTTCTTTACCCTCTG
*TNFA*	GTCTACTCCCAGGTTCTCTT	GGTTGACTTTCTCCTGGTATG
IL1B	TCACAAGCAGAGCACAAG	GAAACAGTCCAGCCCATAC

## Data Availability

The data presented in this study is available on request from the corresponding author.

## References

[1] Cecconi M., Evans L., Levy M., Rhodes A. (2018). Sepsis and septic shock. Lancet.

[2] Hu D., Sheeja Prabhakaran H., Zhang Y.Y., Luo G., He W., Liou Y.C. (2024). Mitochondrial dysfunction in sepsis: Mechanisms and therapeutic perspectives. Crit. Care.

[3] Lai B., Gu M., Sun J., Zhang J. (2025). Targeting mitochondria: Unveiling novel therapeutic frontiers in sepsis. Int. Immunopharmacol..

[4] Sousa A.A.P., Chaves L.D.S., Tarso Facundo H. (2025). Mitochondrial electron transport chain disruption and oxidative stress in lipopolysaccharide-induced cardiac dysfunction in rats and mice. Free Radic. Res..

[5] Oliveira T.S., Santos A.T., Andrade C.B.V., Silva J.D., Blanco N., Rocha N.N., Woyames J., Silva P.L., Rocco P.R.M., da-Silva W.S. (2021). Sepsis Disrupts Mitochondrial Function and Diaphragm Morphology. Front. Physiol..

[6] Klawitter F., Ehler J., Bajorat R., Patejdl R. (2023). Mitochondrial Dysfunction in Intensive Care Unit-Acquired Weakness and Critical Illness Myopathy: A Narrative Review. Int. J. Mol. Sci..

[7] Zong Y., Li H., Liao P., Chen L., Pan Y., Zheng Y., Zhang C., Liu D., Zheng M., Gao J. (2024). Mitochondrial dysfunction: Mechanisms and advances in therapy. Signal Transduct. Target. Ther..

[8] Crouser E.D. (2005). Respiratory failure during critical illness: Are mitochondria to blame?. Am. J. Respir. Crit. Care Med..

[9] Dridi H., Yehya M., Barsotti R., Liu Y., Reiken S., Azria L., Yuan Q., Bahlouli L., Soni R.K., Marks A.R. (2023). Aberrant mitochondrial dynamics contributes to diaphragmatic weakness induced by mechanical ventilation. PNAS Nexus.

[10] Cao L., Li Y., Smirnov A., Voshtani R., Wang T., Shao C., Candi E., Melino G., Shi Y., Fang J. (2025). PGC-1alpha: Key regulator of mitochondrial biogenesis and cellular differentiation in metabolic and regenerative tissues. Cell Biosci..

[11] Yashooa R.K., Duranti E., Conconi D., Lavitrano M., Mustafa S.A., Villa C. (2025). Mitochondrial microRNAs: Key Drivers in Unraveling Neurodegenerative Diseases. Int. J. Mol. Sci..

[12] Rittirsch D., Huber-Lang M.S., Flierl M.A., Ward P.A. (2009). Immunodesign of experimental sepsis by cecal ligation and puncture. Nat. Protoc..

[13] Yang X., Lu G.P., Cai X.D., Lu Z.J., Kissoon N. (2019). Alterations of complex IV in the tissues of a septic mouse model. Mitochondrion.

[14] Bodine S.C., Latres E., Baumhueter S., Lai V.K., Nunez L., Clarke B.A., Poueymirou W.T., Panaro F.J., Na E., Dharmarajan K. (2001). Identification of ubiquitin ligases required for skeletal muscle atrophy. Science.

[15] Cai Y., Yu X., Hu S., Yu J. (2009). A brief review on the mechanisms of miRNA regulation. Genom. Proteom. Bioinform..

[16] Yu Y., Chu W., Chai J., Li X., Liu L., Ma L. (2016). Critical role of miRNAs in mediating skeletal muscle atrophy (Review). Mol. Med. Rep..

[17] Mooren F.C., Viereck J., Kruger K., Thum T. (2014). Circulating microRNAs as potential biomarkers of aerobic exercise capacity. Am. J. Physiol. Heart Circ. Physiol..

[18] Baggish A.L., Park J., Min P.K., Isaacs S., Parker B.A., Thompson P.D., Troyanos C., D’Hemecourt P., Dyer S., Thiel M. (2014). Rapid upregulation and clearance of distinct circulating microRNAs after prolonged aerobic exercise. J. Appl. Physiol. 1985.

[19] Sun Y., Cui D., Zhang Z., Zhang Q., Ji L., Ding S. (2016). Voluntary wheel exercise alters the levels of miR-494 and miR-696 in the skeletal muscle of C57BL/6 mice. Comp. Biochem. Physiol. B Biochem. Mol. Biol..

[20] Mendez-Garcia A., Garcia-Mendoza M.A., Zarate-Peralta C.P., Flores-Perez F.V., Carmona-Ramirez L.F., Pathak S., Banerjee A., Duttaroy A.K., Paul S. (2025). Mitochondrial microRNAs (mitomiRs) as emerging biomarkers and therapeutic targets for chronic human diseases. Front. Genet..

[21] Afzal M., Greco F., Quinzi F., Scionti F., Maurotti S., Montalcini T., Mancini A., Buono P., Emerenziani G.P. (2024). The Effect of Physical Activity/Exercise on miRNA Expression and Function in Non-Communicable Diseases—A Systematic Review. Int. J. Mol. Sci..

[22] Chavez-Guevara I.A., Vazquez-Lorente H., Herrera-Quintana L., Rubio-Valles M., Lopez L.C., Plaza-Diaz J., Amaro-Gahete F.J. (2026). Noncoding RNA molecules mediating skeletal muscle mitochondrial function and their potential applications in exercise molecular physiology: A systematic review. Am. J. Physiol. Cell Physiol..

[23] Simonson S.G., Welty-Wolf K., Huang Y.T., Griebel J.A., Caplan M.S., Fracica P.J., Piantadosi C.A. (1994). Altered mitochondrial redox responses in gram negative septic shock in primates. Circ. Shock.

[24] Takahashi Y., Morisawa T., Okamoto H., Nakanishi N., Matsumoto N., Saitoh M., Takahashi T., Fujiwara T. (2023). Diaphragm Dysfunction and ICU-Acquired Weakness in Septic Shock Patients with or without Mechanical Ventilation: A Pilot Prospective Observational Study. J. Clin. Med..

[25] Carre J.E., Orban J.C., Re L., Felsmann K., Iffert W., Bauer M., Suliman H.B., Piantadosi C.A., Mayhew T.M., Breen P. (2010). Survival in critical illness is associated with early activation of mitochondrial biogenesis. Am. J. Respir. Crit. Care Med..

[26] Mattick J.S., Yang Q., Orman M.A., Ierapetritou M.G., Berthiaume F., Androulakis I.P. (2012). Long-term gene expression profile dynamics following cecal ligation and puncture in the rat. J. Surg. Res..

[27] Merrell A.J., Kardon G. (2013). Development of the diaphragm—a skeletal muscle essential for mammalian respiration. FEBS J..

[28] Callahan L.A., Supinski G.S. (2005). Sepsis induces diaphragm electron transport chain dysfunction and protein depletion. Am. J. Respir. Crit. Care Med..

[29] Powers S.K., Wiggs M.P., Sollanek K.J., Smuder A.J. (2013). Ventilator-induced diaphragm dysfunction: Cause and effect. Am. J. Physiol. Regul. Integr. Comp. Physiol..

[30] Gill L.C., Ross H.H., Lee K.Z., Gonzalez-Rothi E.J., Dougherty B.J., Judge A.R., Fuller D.D. (2014). Rapid diaphragm atrophy following cervical spinal cord hemisection. Respir. Physiol. Neurobiol..

[31] Levine S., Nguyen T., Taylor N., Friscia M.E., Budak M.T., Rothenberg P., Zhu J., Sachdeva R., Sonnad S., Kaiser L.R. (2008). Rapid disuse atrophy of diaphragm fibers in mechanically ventilated humans. N. Engl. J. Med..

[32] Goligher E.C., Dres M., Fan E., Rubenfeld G.D., Scales D.C., Herridge M.S., Vorona S., Sklar M.C., Rittayamai N., Lanys A. (2018). Mechanical Ventilation-induced Diaphragm Atrophy Strongly Impacts Clinical Outcomes. Am. J. Respir. Crit. Care Med..

[33] Grosu H.B., Ost D.E., Lee Y.I., Song J., Li L., Eden E., Rose K. (2017). Diaphragm Muscle Thinning in Subjects Receiving Mechanical Ventilation and Its Effect on Extubation. Respir. Care.

[34] Ebihara S., Hussain S.N., Danialou G., Cho W.K., Gottfried S.B., Petrof B.J. (2002). Mechanical ventilation protects against diaphragm injury in sepsis: Interaction of oxidative and mechanical stresses. Am. J. Respir. Crit. Care Med..

[35] Supinski G.S., Schroder E.A., Wang L., Morris A.J., Callahan L.A.P. (2021). Mitoquinone mesylate (MitoQ) prevents sepsis-induced diaphragm dysfunction. J. Appl. Physiol. 1985.

[36] Jung B., Nougaret S., Conseil M., Coisel Y., Futier E., Chanques G., Molinari N., Lacampagne A., Matecki S., Jaber S. (2014). Sepsis is associated with a preferential diaphragmatic atrophy: A critically ill patient study using tridimensional computed tomography. Anesthesiology.

[37] Supinski G.S., Morris P.E., Dhar S., Callahan L.A. (2018). Diaphragm Dysfunction in Critical Illness. Chest.

[38] Gunst J., Umpierrez G.E., Van den Berghe G. (2024). Managing blood glucose control in the intensive care unit. Intensive Care Med..

[39] Peeters B., Langouche L., Van den Berghe G. (2017). Adrenocortical Stress Response during the Course of Critical Illness. Compr. Physiol..

[40] Vanhorebeek I., Langouche L., Van den Berghe G. (2006). Endocrine aspects of acute and prolonged critical illness. Nat. Clin. Pract. Endocrinol. Metab..

[41] Fu W., Guan L., Liu Q., Xie Z., You J., Chen R. (2025). Ventilator-induced diaphragmatic dysfunction: Pathophysiology, monitoring and advances in potential treatment and prevention. Eur. Respir. Rev..

